# Multiple genotype–phenotype association study reveals intronic variant pair on *SIDT2* associated with metabolic syndrome in a Korean population

**DOI:** 10.1186/s40246-018-0180-4

**Published:** 2018-11-01

**Authors:** Sanghoon Moon, Young Lee, Sungho Won, Juyoung Lee

**Affiliations:** 1 Division of Genome Research, Center for Genome Science, Korea National Institute of Health, Cheongju, Chungcheongbuk-do 28159 South Korea; 2 Veterans Medical Research Institute, Veterans Health Service Medical Center, Seoul, 05368 South Korea; 3 Department of Public Health Science, Seoul National University, Seoul, 08826 South Korea

**Keywords:** Multiple variants, Multiple traits, Metabolic syndrome, 11q23.3, *SIDT2*

## Abstract

**Background:**

Metabolic syndrome is a risk factor for type 2 diabetes and cardiovascular disease. We identified common genetic variants that alter the risk for metabolic syndrome in the Korean population. To isolate these variants, we conducted a multiple-genotype and multiple-phenotype genome-wide association analysis using the family-based quasi-likelihood score (MFQLS) test. For this analysis, we used 7211 and 2838 genotyped study subjects for discovery and replication, respectively. We also performed a multiple-genotype and multiple-phenotype analysis of a gene-based single-nucleotide polymorphism (SNP) set.

**Results:**

We found an association between metabolic syndrome and an intronic SNP pair, rs7107152 and rs1242229, in *SIDT2* gene at 11q23.3. Both SNPs correlate with the expression of *SIDT2* and *TAGLN*, whose products promote insulin secretion and lipid metabolism, respectively. This SNP pair showed statistical significance at the replication stage.

**Conclusions:**

Our findings provide insight into an underlying mechanism that contributes to metabolic syndrome.

**Electronic supplementary material:**

The online version of this article (10.1186/s40246-018-0180-4) contains supplementary material, which is available to authorized users.

## Introduction

Metabolic syndrome is a cluster of metabolic risk factors for cardiovascular disease and type 2 diabetes that are attributable to both genetic and environmental factors [1–3]. The National Cholesterol Education Program’s Adult Treatment Panel III report (2001) defined metabolic syndrome as a combination of components such as high blood pressure, elevated fasting plasma glucose, high serum triglycerides, and abnormal low-density lipoprotein (LDL) and high-density lipoprotein (HDL) cholesterol levels [4]. Because of the fast-growing economy and rapid industrialization of Korea, metabolic syndrome is likely to become a major public health problem [5].

Many genetic variants that are associated with metabolic syndrome have been identified by genome-wide association studies (GWASs). However, because these known genetic variants account for only a fraction of the heritability of metabolic syndrome, the genetic determinants of this condition remain undefined [6, 7]. Many common variants with very small effect sizes that are widely distributed across the genome cannot be identified by the use of traditional GWAS cutoffs [8]. Experimental sample sizes must be large because the statistical power to detect associations between DNA variants and a trait depends on the sample size [9]. However, the cost and difficulties of sample collection inhibit the ability to continuously increase the sample size of GWASs [10]. Joint analysis approaches that analyze multiple genotypes and phenotypes have shown improved ability to detect variants relative to single-variant association analyses of the same-size sample [11–13]. A statistical approach called the MFQLS (http://healthstat.snu.ac.kr/software/mfqls/) test enables the estimation of the genetic relation matrix from population-based samples [14]. From the results of a single test for association with a set of traits, multiple genotype–multiple phenotype analysis reduces the number of tests and mitigates the multiple testing issues, resulting in increased statistical power [13, 14]. This approach identifies genetic variants that have pleiotropic effects for metabolic syndrome and other diseases.

We conducted a multiple single nucleotide polymorphism (SNP)–multiple trait analysis to identify genetic variants associated with metabolic syndrome by utilizing 10,049 samples from Korean subjects. After the multiple-genotype and multiple-phenotype analysis (multi-SNP–multi-trait analysis), 27 SNP pairs were associated with metabolic syndrome in the discovery stage and successfully replicated. Of those pairs, from the joint analysis of a single genotype and multiple phenotypes (single-SNP–multi-trait analysis), we found that three SNP pairs in the respective genes *SIDT2*, *UBASH3B*, and *CUX2* were significant in the multi-SNP–multi-trait analysis but not significant in the single-SNP–multi-trait analysis. *SIDT2* was not previously reported in the NHGRI-EBI GWAS Catalog (https://www.ebi.ac.uk/gwas/) [15] and was significant in the gene-based SNP set and multi-trait analysis. The Genotype-Tissue Expression (GTEx) database shows rs7107152 and rs1242229 on *SIDT2* correlates with *SIDT2* and *TAGLN* expression (https://www.gtexportal.org/home/) [16]. Our findings support the effectiveness of the multi-SNP–multi-trait analysis to identify new susceptible loci in complex diseases.

## Results

### Multi-SNP-multi-trait analysis

We conducted the multi-SNP–multi-trait genome-wide analysis of metabolic syndrome using 10,049 samples from Korean subjects. We considered six quantitative components of metabolic syndrome: systolic blood pressure (SBP), diastolic blood pressure (DBP), high-density lipoprotein (HDL), fasting plasma glucose (FPG), triglyceride, and waist circumference. Through the multi-SNP–multi-trait genome-wide analysis, adjusted for age and sex, 27 SNP pairs satisfied a Bonferroni-adjusted *P* value threshold of *P* < 0.05 (*P* = 1.45 × 10^−7^) and were successfully replicated (Tables 1 and 2). All but four of the mapped genes (*SIK3*, *SIDT2*, *UBASH3B*, and *CUX2*) had been previously reported to be associated with metabolic syndrome, as indicated by a keyword search of “metabolic syndrome” in the GWAS catalog (Tables 1 and 2).

**Table 1:** Twenty-four SNP pairs that were significant in both single- and multi-SNP–multi-trait analyses

SNP pair	Gene	Publication of mapped gene in GWAS catalog	*P* value from a single SNP-multi trait analysis			*P* value from multi SNP-multi trait analysis (A Bonferroni-adjusted *P* value = 1.45 × 10^−7^)		
			Discovery	Replication	Meta *P* value	Discovery	Replication	Meta *P* value
rs2293571, **rs780094**	*GCKR*	[6, 19, 20, 36]	2.39E-03, 2.55E-12	9.46E-02, 1.85E-06	2.12E-03, 1.93E-16	4.98E-14	6.21E-05	1.28E-16
**rs780094**, **rs780092**	*GCKR*	[6, 19, 20, 36]	2.55E-12, 2.08E-11	1.85E-06, 1.36E-02	1.93E-16, 8.46E-12	2.02E-14	5.62E-05	4.80E-17
**rs780092**, rs8179252	*GCKR*	[6, 19, 20, 36]	2.08E-11, 1.93E-03	1.36E-02, 1.26E-02	8.46E-12, 2.83E-04	1.02E-13	4.17E-05	1.74E-16
rs263, rs271	*LPL*	[6, 19, 37]	2.09E-10, 6.96E-11	1.40E-05, 1.57E-05	1.01E-13, 3.87E-14	2.30E-09	2.43E-05	1.76E-12
rs12545984, **rs10503669**	*LPL-SLC18A1*	[6, 19, 37]	2.43E-01, 1.74E-15	2.24E-01, 9.06E-11	2.13E-01,9.16E-24	1.81E-14	9.20E-10	8.90E-22
**rs10503669, rs17410962**	*LPL-SLC18A1*	[6, 19, 37]	1.74E-15, 1.85E-16	9.06E-11, 4.76E-11	9.16E-24, 5.37E-25	3.35E-14	5.28E-09	9.04E-21
rs17489282, rs4922117	*LPL-SLC18A1*	[6, 19, 37]	4.80E-12, 1.09E-12	2.82E-07, 1.82E-07	5.70E-17, 8.74E-18	3.35E-11	6.59E-06	8.18E-15
**rs765547,** rs11986942	*LPL-SLC18A1*	[6, 19, 37]	7.30E-13, 1.54E-12	3.55E-07, 1.46E-07	1.13E-17, 9.88E-18	1.79E-11	2.43E-07	1.78E-16
rs11986942, rs1837842	*LPL-SLC18A1*	[6, 19, 37]	1.54E-12, 2.16E-12	1.46E-07, 3.57E-07	9.88E-18, 3.29E-17	1.21E-10	1.24E-06	5.62E-15
rs1837842, rs1919484	*LPL-SLC18A1*	[6, 19, 37]	2.16E-12, 1.57E-12	3.57E-07, 4.14E-07	3.29E-17, 2.79E-17	4.43E-10	3.19E-05	4.65E-13
rs1919484, rs7461115	*LPL-SLC18A1*	[6, 19, 37]	1.57E-12, 1.77E-12	4.14E-07, 2.61E-07	2.79E-17, 2.00E-17	8.62E-11	1.68E-05	5.09E-14
rs7013777, rs4442164	*LPL-SLC18A1*	[6, 19, 37]	7.61E-13, 1.08E-01	2.12E-07, 3.63E-01	7.14E-18, 1.66E-01	1.40E-16	1.09E-07	8.17E-22
rs4442164, **rs4244457**	*LPL-SLC18A1*	[6, 19, 37]	1.08E-01, 3.42E-14	3.63E-01, 4.04E-06	1.66E-01, 6.14E-18	1.39E-15	1.50E-05	9.66E-19
**rs4244457,** rs4449813	*LPL-SLC18A1*	[6, 19, 37]	3.42E-14, 3.20E-01	4.04E-06, 2.28E-01	6.14E-18, 2.64E-01	3.54E-14	1.69E-05	2.57E-17
**rs12686004, rs3905000**	*ABCA1*	[6]	1.25E-09, 6.74E-02	1.13E-02, 7.66E-01	3.67E-10, 2.05E-01	3.08E-10	4.22E-02	3.39E-10
rs481843, rs486394	*LOC101929011*	[38]	2.40E-06, 4.03E-08	6.85E-03, 1.79E-03	3.11E-07, 1.76E-09	3.33E-08	1.85E-02	1.37E-08
rs180344, **rs11216126**	*LOC101929011-BUD13*	[19, 38]	9.22E-02, 3.70E-20	9.04E-01, 3.25E-08	2.90E-01, 7.57E-26	5.54E-20	5.66E-07	1.87E-24
**rs6589566, rs603446**	*ZPR1*	[6]	8.27E-26, 9.02E-14	2.69E-12, 1.15E-05	1.90E-35, 4.40E-17	1.03E-28	3.51E-12	3.32E-38
rs11600380, rs6589567	*APOA5-APOA4*	[38]	8.27E-06, 1.14E-06	2.95E-02, 1.82E-05	3.96E-06, 5.31E-10	6.60E-09	2.32E-05	4.67E-12
rs12279433, rs11827828	*SIK3*	–	2.29E-07, 3.35E-05	2.85E-05, 6.27E-01	1.75E-10, 2.47E-04	1.35E-09	6.84E-04	2.65E-11
rs11827828, rs2044426	*SIK3*	–	3.35E-05, 2.23E-07	6.27E-01, 9.96E-06	2.47E-04, 6.18E-11	1.30E-09	2.84E-04	1.09E-11
rs10892044, rs11216186	*SIK3*	–	7.85E-05, 1.14E-06	6.58E-01, 5.91E-05	5.62E-04, 1.65E-09	1.64E-08	1.40E-03	5.85E-10
**rs2074356,** rs11066194	*HECTD4*	[21]	8.85E-24, 4.83E-02	9.27E-07, 4.76E-01	5.58E-28, 1.10E-01	1.19E-20	2.49E-05	1.70E-23
rs11631342, **rs6494005**	*LIPC*	[6, 19]	6.22E-08, 1.41E-10	3.16E-01, 1.51E-02	3.68E-07, 5.93E-11	1.63E-14	1.24E-02	7.51E-15

SNPs that are listed in GWAS catalog are shown in boldface

**Table 2:** Three SNP pairs that were significant only in the multi-SNP–multi-trait analysis

SNP pair	CHR	Position (hg19)	Gene	Annotation	Single-multi *P* value			Multi-multi *P* value (A Bonferroni-adjusted *P* value of discovery stage = 1.45 × 10^−7^)		
					Discovery	Replication	Meta *P* value	Discovery	Replication	Meta *P* value
rs7107152	11	117,056,080	*SIDT2*	Intronic	2.59E-01	8.43E-01	5.51E-01	1.40E-08	2.32E-03	8.17E-10
rs1242229	11	117,062,370	*SIDT2*	Intronic	5.39E-03	7.68E-02	3.64E-03			
rs10892876	11	122,540,281	*UBASH3B*	Intronic	2.93E-01	1.27E-01	1.60E-01	3.34E-12	3.82E-03	4.21E-13
rs12290043	11	122,540,528	*UBASH3B*	Intronic	1.62E-01	1.46E-01	1.12E-01			
rs886126	12	111,679,214	*CUX2*	Intronic	3.51E-01	1.79E-01	2.37E-01	5.09E-13	3.35E-03	5.97E-14
rs2078851	12	111,690,579	*CUX2*	Intronic	7.31E-05	6.63E-02	6.42E-05			

*Single-multi P value P* value from a single-SNP–multi-trait association analysis, *multi-multi P value P* value from a multi-SNP–multi-trait association analysis, *CHR* chromosome, *Meta P value P* value from meta-analysis

### Single SNP set-multi-trait analysis

The relation of the individual SNPs, including those on *SIK3*, *SIDT2*, *UBASH3B*, and *CUX2*, to metabolic syndrome was further examined by single-SNP–multi-trait analysis, adjusted for age and sex. Intronic SNPs on *SIK3* showed genome-wide significance in single-SNP–multi-trait analysis (Table 1), but SNPs on *SIDT2*, *UBASH3B*, and *CUX2* not identified by this approach (Table 2).

### Gene-based SNP set-multi-trait analysis

We conducted gene-based SNP set–multi-trait analysis on 14,475 SNP sets. The mean and median numbers of SNPs in a set were 6.895 and 3, respectively (Additional file 1: Table S1). Table 3 shows the gene-based test results. Three genes reached a Bonferroni-adjusted *P* value threshold (*P* < 3.45 × 10^−6^). All three genes satisfied statistical significance in the meta-analysis. The number of SNPs in three genes ranges from 3 to 14. SNP sets in two genes (*PAFAH1B2* and *SIDT2*) showed significant *P* values of < 3.45 × 10^− 6^ in the gene-based test and reached a nominal *P* value threshold of < 0.05 in the replication stage. Although the results of tests in which 14 SNPs were utilized in *CUX2* were significant in the discovery stage, there was only a suggested *P* value of 0.06 in the replication analysis (Table 3). Table 3 also shows the SNPs used in each SNP set.

**Table 3:** Results from a gene-based SNP set and multi-trait analysis

Gene	The number of SNP	Chromosome	Position (hg19)	Maximum *r*^2^	*P* value (a Bonferroni-adjusted *P* value of discovery stage *P* = 3.45 × 10^−6^)		
					Discovery	Replication	Meta *P* value
*PAFAH1B2*	rs12420127	11	117,035,319	0.466	4.00E-07	3.99E-03	3.39E-08
	rs10790175		117,034,729				
	rs10892082		117,039,325				
*SIDT2*	rs2269399	11	117,066,353	0.456	2.31E-07	3.83E-03	1.93E-08
	rs1242229		117,062,370				
	rs1784042		117,065,476				
	rs7107152		117,056,080				
*CUX2*	rs7952972	12	111,646,519	0.561	3.28E-10	6.34E-02	5.32E-10
	rs886126		111,679,214				
	rs7300082		111,737,115				
	rs4766553		111,634,281				
	rs1265566		111,716,376				
	rs9783423		111,639,456				
	rs7398833		111,786,892				
	rs16941414		111,779,792				
	rs6489979		111,614,736				
	rs16941284		111,610,723				
	rs16941319		111,646,853				
	rs11065851		111,723,739				
	rs756825		111,598,202				
	rs7300860		111,754,597				

### Expression QTL pattern of identified SNP pair

To examine the correlation between two SNPs (rs7107152 and rs1242229) on the *SIDT2* gene and gene expression, we utilized three online resources: the Genotype-Tissue Expression (GTEx) project (https://www.gtexportal.org/home/) [16], the NESDA NTR Conditional Expression Quantitative Trait Loci (eQTL) catalog (https://eqtl.onderzoek.io/), and RegulomeDB (http://www.regulomedb.org/) [17]. All three databases showed that the two SNPs correlated with *SIDT2* and *TAGLN* expressions. The GTEx project showed that for rs7107152, the statistical significance of *SIDT2* and *TAGLN* expression in whole blood was *P* = 3.89 × 10^−14^ and *P* = 3.59 × 10^− 47^, respectively (Additional file 1: Figure S1A), and for rs1242229, the *P* values were 3.64 × 10^−13^ and 4.78 × 10^−12^, respectively (Additional file 1: Figure S1B) [16]. Additional file 1: Figure S1C–F show gene expression patterns by genotype for the two SNPs. The eQTL catalog showed similar gene expression patterns for the two SNPs (Additional file 1: Figure S2). The SNP rs1242229 was mainly expressed in *SIDT2* and *TAGLN*, whereas the SNP rs7107152 showed gene expression patterns not only in *SIDT2* and *TAGLN* but also in *PCSK7* and *PAFAH1B2* (Additional file 1: Figure S2). Additional file 1: Figure S3 shows the gene expression pattern for rs1242229 in RegulomeDB [17].

### Correlation test with previously reported SNPs

**Fig. 1:**
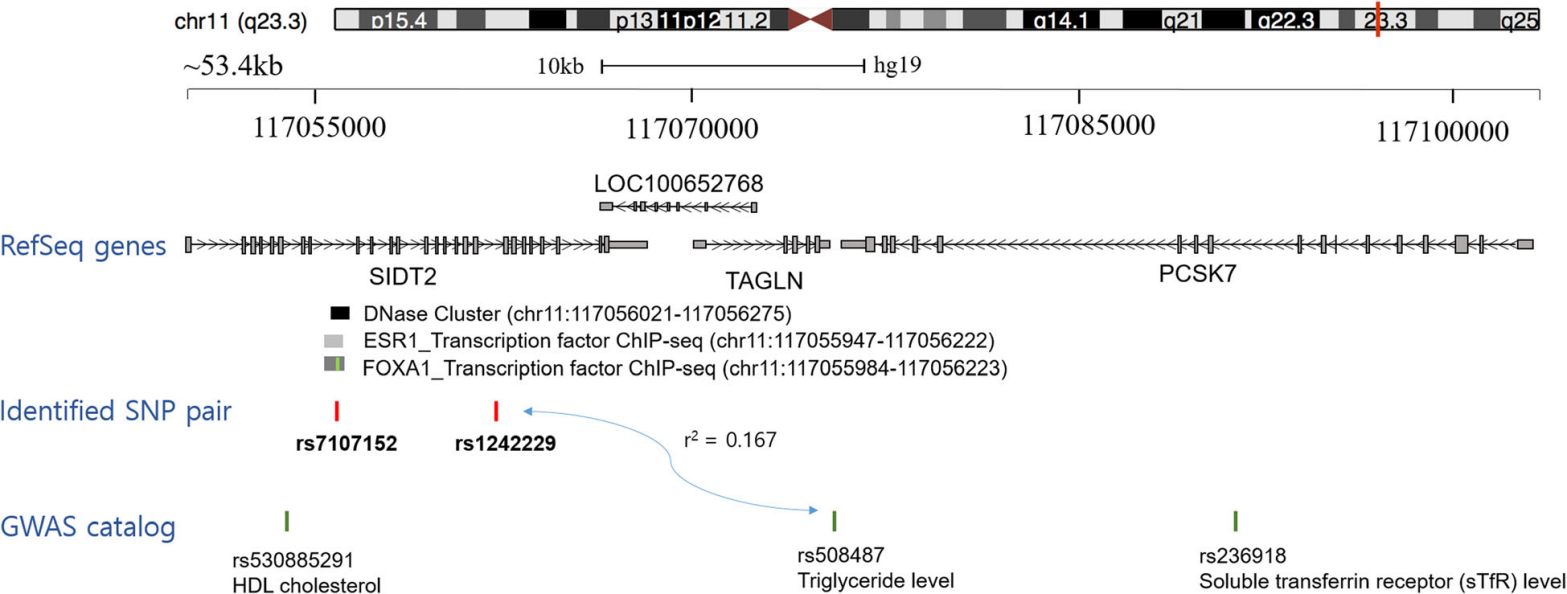
Overview of GWAS results showing the significant SNP pair at 11q23.3. The SNP pair is marked by the red vertical bars. Green vertical bars depict reported SNPs in the GWAS catalog

To calculate the correlation of the identified SNPs (rs7107152 and rs1242229) in the current study and nearby SNPs reported in the GWAS catalog (intronic variant rs530885291, associated with HDL cholesterol level, and intergenic variant rs508487, associated with triglyceride level), we conducted a pairwise linkage disequilibrium (LD) analysis utilizing the SNP Annotation and Proxy Search (SNAP) server (https://www.broadinstitute.org/snap/snap) [18]. The pairwise LD analysis showed that rs1242229 had a squared correlation of *r*^2^ = 0.167 with rs508487 (Fig. 1). The correlation score between rs7107152 and rs530885291, however, was lower than the cutoff score.

### SNP pair-single trait analysis

A radar chart shows the results of the rs7107152 and rs1242229 SNP metabolic syndrome-component trait association pair analysis (Additional file 1: Figure S4). The SNP pair correlated with HDL (*P* = 5.87 × 10^−5^) and triglycerides (*P* = 1.67 × 10^−9^). Moreover, it showed a suggestive association with diastolic blood pressure (*P* = 0.07). But it did not correlate with waist circumference (*P* = 0.75), fasting plasma glucose (*P* = 0.55), systolic blood pressure (*P* = 0.29).

## Discussion

Utilizing the MFQLS test, we found 27 SNP pairs associated with metabolic syndrome based on multi-SNP–multiple continuous phenotypes. Our keyword search for the term “metabolic syndrome” found single-variant association results in the GWAS catalog. The association of metabolic syndrome or metabolic syndrome-component traits with 21 SNP pairs was found in the GWAS catalog [15], whereas the association of 6 SNP pairs on the genes SIK3, SIDT2, UBASH3B, and CUX2 was not found (Table 1). Not only lipid loci but also insulin-associated loci associated with metabolic syndrome. However, most of the variants identified were present in known lipid loci. In contrast, a relatively small number of variants were in other metabolic syndrome-component traits. For example, genetic variants such as rs780092 and rs780094 mapped on the *GCKR* gene were susceptible variants relative to total cholesterol, fasting glucose level, and lipid metabolism phenotypes [6, 19, 20]. An SNP from the pairs identified here, rs2074356, which mapped on the *HECTD4* gene, was previously associated with the glycemic trait [21]. Six of the SNP pairs identified have shown an association with the lipid trait–associated LD region spanning *BUD13*-*ZNF259*, *APOA5*-*A4*-*C3*-*A1*, and *SIK3* [6, 13, 19, 22]. Povel et al. [22] reported a systematic review of genetic variants and metabolic syndrome. They suggested that although disturbances in metabolic syndrome-component traits have been proposed to activate metabolic syndrome, most SNPs associated with metabolic syndrome are in genes involved in lipid metabolism [23]. Moreover, Kristiansson et al. [6] showed that genes from the lipid metabolism pathway are factors in metabolic syndrome. However, these investigators found little evidence associated with other metabolic syndrome-component traits such as hypertension and glucose intolerance [6]. Our results were consistent with previous studies.

Through the single-SNP–multi-trait analysis, we identified four SNP pairs that were associated with metabolic syndrome but were not in the GWAS catalog. Intronic SNPs on the *SIK3* gene showed genome-wide significance in the single-SNP–multi-trait analysis (Table 1). SNPs on the *UBASH3B* and *CUX2* genes could not be identified by single-SNP–multi-trait analysis (Table 2), whereas the association of *UBASH3B* and *CUX2* with metabolic syndrome-component traits was reported in the GWAS catalog. For example, two variants, rs7128198 on the 5′-untranslated region and rs7941030 upstream of *UBASH3B*, were associated with total cholesterol and HDL level, respectively [24, 25]. The intergenic variant rs12229654 between *MYL2* and *CUX2* showed a pleiotropic effect associated with metabolic syndrome, HDL, and glycemic traits [21, 26]. An association between cardiovascular disease and rs886126, which also identified in our study, was previously reported [27].

Utilizing multi-SNP–multi-trait analysis, we found an association between the *SIDT2* gene and metabolic syndrome or metabolic syndrome-component traits based on an association between metabolic syndrome and rs7107152 and rs1242229. The SNP rs7107152 is within the DNase cluster in the region of transcription factors such as ESR1 and FOXA1 (Fig. 1). The SNPs rs530885291 and rs508487 showed in the GWAS catalog as proximal to rs7107152 and rs1242229, respectively [15]. However, the pairwise LD score between rs1242229 and rs508487 was low (*r*^2^ = 0.167), indicating little correlation between these two SNPs.

We performed another multi-SNP–multi-trait analysis based on the SNP set test (gene-based test). SNP sets on the *PAFAH1B2*, *SIDT2*, and *CUX2* genes showed significant *P* values in the gene-based test (a Bonferroni-adjusted *P* value threshold is *P* = 3.45 × 10^−6^). *PAFAH1B2* and *SIDT2* reached a replication *P* value of **<** 0.05. The SNP set on *CUX2* suggested a *P* value of 0.06 in the replication stage (Table 3); a larger sample size might have resulted in a significant *P* value. The SNP set rs7107152 and rs1242229 on *SIDT2* was also significant, supporting the association of *SIDT2* with metabolic syndrome.

We found evidence from the three online databases that rs7107152 and rs1242229, which are in an intron of *SIDT2*, correlated with the expression of *SIDT2* and *TAGLN* (Additional file 1: Figure S1). Two recent eQTL studies provide further evidence that these two SNPs alter gene expression that is relevant to metabolic syndrome-component traits [28, 29]. From the eQTL analysis between SNP and whole-blood gene expression in 5,257 Framingham Heart Study participants, rs1242229 was reported as a proxy SNP for altered *SIDT2* and *TAGLN* expression in triglyceride [28]. Huan et al. [29] investigated *cis* and *trans* eQTL by utilization of the human whole-blood transcriptome data from Framingham Heart Study pedigrees. The SNP rs7107152 also altered *SIDT2* expression in triglyceride [8]. The radar chart shows that these variants increase HDL and triglyceride levels among metabolic syndrome-component traits, supporting the hypothesis that genes are a key factor in the link between lipid metabolism and metabolic syndrome (Additional file 1: Figure S4) [6, 22]. We conferred that lipid traits such as HDL and TG have the greatest impact on metabolic syndrome, but weak associations such as DBP may also have an impact. However, due to the limitations of current research, the potential weak association must interpret carefully. The eQTL SNPs correlated with *SIDT2* and *TAGLN* expression enriched in the 100-kb region (117000–117,100 kb) around *SIDT2* and *TAGLN* (Additional file 1: Figure S5).

SID1 transmembrane family member 2 (SIDT2) is a lysosomal integral membrane protein that promotes insulin secretion [30]. Recently, Gao et al. [31] described its activity in insulin secretion. *Sidt2*^*−/−*^ mice exhibit weight loss and increased fasting glucose levels and impaired glucose tolerance. These investigators identified mouse SIDT2 function in lipid metabolism. SIDT2-deficient mice have increased serum triglyceride [32]. TAGLN (Transgelin, sm22α) is an actin-binding protein expressed in smooth muscle cells [33]. Yang et al. [34] revealed that the most enriched pathways caused by SM22α knockout in mice were lipid metabolism, inflammation, and hematopoiesis. We hypothesize that the SNP pair associated with metabolic syndrome activate expression of the genes *SIDT2* and *TAGLN*. A difference in gene expression might inhibit insulin secretion, lipid metabolism, and adipogenesis, resulting in metabolic syndrome (Additional file 1: Figure S6). Although evidence supports the association between the identified SNPs and metabolic syndrome, functional investigations of the SNPs are needed. However, functional investigation using identified 27 SNP pairs in non-coding regions is limited. Given additional information such as imputed SNPs, a metabolic syndrome-associated genetic variant in coding regions may be detected by the statistical test used in the present study.

## Conclusions

Our results show that a multi-SNP–multi-trait analysis is an efficient approach for finding variants that have not previously been isolated from single-variant–multi-trait analysis. The advantage of this approach is an increase in statistical power that results from considering the combined effects of two variants. Because *P* values of identified variants such as rs7107152 and rs1242229 did not reach statistical significance, these variants cannot be identified by single-variant analysis of the same sample size. Although previously identified variants are in lipid loci, these variants and their mapped genes are not reported in metabolic syndrome. Our findings provide insight into the genetic variant contribution to metabolic syndrome.

## Methods

### Study design and participants

**Fig. 2:**
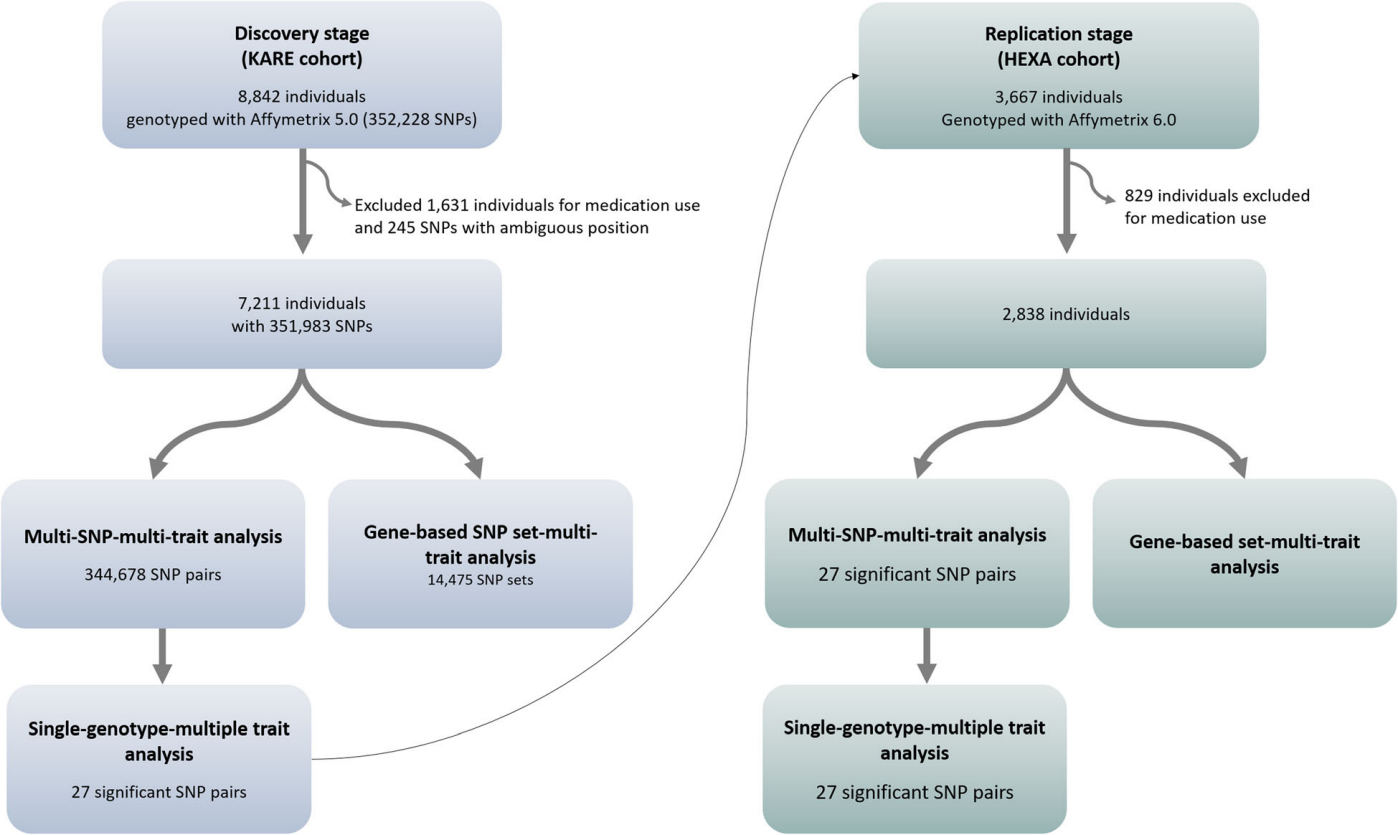
Schematic diagram of the current study. KARE, Korean Association REsource Project; HEXA, Health Examinees

To identify susceptible variant sets associated with metabolic syndrome, we conducted a multi-SNP–multi-trait analysis through the two stages: discovery and replication analysis (Fig. 2). For the discovery stage, we used genome data from the Ansan/Ansung cohort in the Korean Genome Epidemiology Study, which is known as the Korea Association REsource (KARE) project [35]. In the subsequent replication stage, we used the Health Examinees (HEXA) study cohort, data from which were also used in the GWASs [26, 27]. All participants were between 40 and 69 years of age. Informed consent was obtained from all participants. This study was approved by the ethical committee of the Korea Centers for Disease Control and Prevention Institutional Review Board. Detailed demographic information of participants is shown in Table 4.

**Table 4:** Characteristics of the study participants

	KARE			HEXA	
	Case (*n* = 1328)	Control (*n* = 5870)	Not determined (*n* = 13)	Case (*n* = 309)	Control (*n* = 2529)
Age	53.9 (8.65)	50.52 (8.62)	61.31 (8.02)	54 (7.92)	51.48 (7.88)
Sex (m/f)	561/767	3000/2870	4/9	184/125	1039/1490
SBP	128.15 (18.13)	112.36 (15.52)	128.36 (16.07)	132.19 (13.77)	118.68 (13.47)
DBP	81.97 (11.46)	72.32 (10.49)	74.92 (6.43)	83.54 (9.12)	75.39 (9.47)
FPG	94 (29.82)	84.31 (14.79)	80.67 (1.53)	101.49 (25.38)	90.15 (22.3)
Triglyceride	241.35 (134.62)	138.94 (83.58)	180.92 (122.1)	224.38 (134.91)	106.35 (74.49)
log triglyceride	5.38 (0.44)	4.82 (0.44)	5.04 (0.55)	5.28 (0.5)	4.52 (0.52)
HDL	38.35 (6.78)	46.54 (10.06)	42.58 (8.39)	43.5 (8.88)	57 (13.16)
WC	90.33 (7.01)	79.98 (7.82)	87.27 (7.97)	90.69 (6.86)	80.44 (8.13)

*n* sample size; *SBP* systolic blood pressure, *DBP* diastolic blood pressure, *FPG* fasting plasma glucose, *HDL* high-density lipoprotein, *WC* waist circumference

*KARE* Korean Association Resource Project, *HEXA* Health Examinee cohort

Data are shown as the mean (SD)

### Quality control in GWAS

The KARE data consisted of 10,004 samples genotyped with Affymetrix Genome-Wide Human SNP array 5.0 [26, 35]. We selected 8,842 individuals genotyped with 352,228 SNPs from the quality control process. The quality control criteria and process have been described [21, 26, 35]. We used 351,983 SNPs for the multi-SNP–multi-trait analysis after exclusion of 245 SNPs with ambiguous chromosome numbers and positions. We selected 7,211 of 8,842 participants who did not take lipid-lowering or anti-diabetes medication. For the replication analysis, we selected 2,838 of 3,701 participants from the HEXA study after those taking medication were excluded.

### Phenotyping

We considered six quantitative traits as components of metabolic syndrome. We used the National Cholesterol Education Program Expert Panel on Detection, Evaluation, and Treatment of High Blood Cholesterol in Adults (Adult Treatment Panel III) final report guideline as metabolic syndrome definition [4]. For waist circumference, we used modified Asian guidelines, which reduce the limit from > 102 cm to > 90 cm for men and from > 88 cm to > 80 cm for women. Thus, a participant was considered to have metabolic syndrome if he or she had three of the five following features: (1) triglyceride > 150 mg/dL, (2) HDL < 40 mg/dL for men and < 50 mg/dL for women, (3) waist circumference > 90 cm for men and > 80 cm for women, (4) fasting plasma glucose > 110 mg/dL, and (5) blood pressure threshold > 130 mmHg (SBP) and > 85 mmHg (DBP).

### Multiple SNP set–multiple trait association analysis

Previously, Won et al. [14] proposed a statistical method for the joint analysis of multiple phenotypes and genotypes. This method, the MFQLS (http://healthstat.snu.ac.kr/software/mfqls/), can be utilized for both quantitative and dichotomous phenotypes. It can be applied to large-scale, genome-wide association analysis as well as family-based samples. The empirical power test showed that it is statistically more efficient than existing methods. In addition, the genome-wide association analysis of 1,801 individuals with obesity showed that *P* values from the MFQLS were markedly less than those from other methods [14]. In our study, two types of multiple SNP sets, such as paired SNPs and gene-based SNPs having more than three variants, were considered to be multiple genotypes. For the multi-SNP–multi-trait analysis, the MFQLS incorporates both multiple traits and SNPs into a single test statistic. For example, given two traits and two SNPs, the MFQLS tests *H*_0_: *β*_11_ = *β*_12_ = *β*_21_ = *β*_22_ = 0, where *β*_*ij*_ denotes the effect of association between the *i*th SNP and the *jth* trait. The effect of these genotypes and the statistical significance of this effect are greater when multiple genotypes and phenotypes are correlated. Of 351,983 SNPs, 344,677 SNP pairs with MAF > 0.01 were selected (a Bonferroni-adjusted *P* value threshold, *P* = 1.45 × 10^−7^). Additional file 1: Table S2 shows MAF of identified SNPs. We extended the gene-based genome-wide association analysis with multiple traits. To select an SNP set for gene-based analysis, we included common SNPs in the first set on the platform used in the discovery and replication stages. We selected gene-based tag SNPs captured with *r*^2^ ≥ 0.8 by the use of Tagger (http://www.broad.mit.edu/mpg/tagger/) for the second set. Consequently, 14,475 SNP sets were selected for gene-based analysis (a Bonferroni-adjusted *P* value threshold, *P* = 3.45 × 10^−6^). Table 3 and Additional file 1: Table S1 show information about the SNP sets used for the gene-based test.

Two-stage analyses such as estimation of correlation between each SNP and calculation of statistics are used to run the MFQLS.

The Fisher’s combined probability test was used to calculate the meta-analysis from discovery and replication results by application of MADAM in the R package.

### Single-SNP–multi-trait association analysis

To determine whether a significant SNP pair identified in the multi-SNP–multi-trait analysis was still significant if a different approach was used, we performed a joint analysis between single genotype and multiple phenotypes. An SNP and multiple traits were incorporated into the statistics of a single test and three traits were given, *H*_0_: *β*_1_ = *β*_2_ = *β*_3_ = 0 and *H*_1_*:* not *H*_0_, where *β*_*i*_ denotes the effect of association between a SNP and the *ith* trait. Covariates such as age and sex were adjusted in the analysis.

### SNP pair–metabolic syndrome-component trait association analysis

To determine which metabolic syndrome-component traits are related to the SNP pair rs7107152 and rs1242229, we performed an association analysis between the SNP pair and each metabolic syndrome-component trait.

### Additional analyses from online data resources

To examine the expression pattern of each SNP of an identified SNP pair, we utilized three online data resources that provide eQTL information: the portal for GTEx [16], RegulomeDB [17], and the NESDA NTR Conditional eQTL Catalog. SNAP was applied to calculate the correlation between identified SNPs and previously known GWAS SNPs [18].

## Additional files

Additional file 1: Supplementary information. **Figure S1.** Screenshot of GTEx database for significant SNP pair rs7107152 and rs1242229. **Figure S2.** Screenshot of NESDA NTR Conditional eQTL Catalog for rs7107152 and rs1242229. **Figure S3.** Screenshot of RegulomeDB for rs1242229. **Figure S4.** Radar chart showing the result of the rs7107152 and rs1242229 SNP pair–single trait (metabolic syndrome-component trait) association analysis. **Figure S5.** Screenshot from the GTEx database of the eQTL SNP-enriched region around *SIDT2* and *TAGLN* (chr11:117,000,000-117,100,000). Figure S6 Model for how significant SNP pair rs7107152/rs1242229 may affect metabolic syndrome risk. **Table S1.** Characteristics of gene-based SNPs set. **Table S2.** Minor allele frequency of identified SNPs. (PDF 975 kb)

Additional file 2: MFQLS results. (TXT 16795 kb)

## Abbreviations

DBP: Diastolic blood pressure
eQTL: Expression quantitative trait loci
FPG: Fasting plasma glucose
GTEx: Genotype-tissue expression
GWAS: Genome-wide association study
HDL: High-density lipoprotein cholesterol
HEXA: Health Examinees
KARE: Korean Association REsource Project
LDL: Low-density lipoprotein cholesterol
MFQLS: The family-based quasi-likelihood score test
SBP: Systolic blood pressure
SIDT2: SID1 transmembrane family member 2
TAGLN: Transgelin
TC: Total cholesterol
TG: Triglyceride
