# Supplementary material for: Multiple genotype–phenotype association study reveals intronic variant pair on SIDT2 associated with metabolic syndrome in a Korean population
Source: Hum Genomics. 2018 Nov 1;12:48. doi: 10.1186/s40246-018-0180-4 (PMC6211397; doi:10.1186/s40246-018-0180-4)
Supplement: Supplementary file 1 — Supplementary information. Figure S1. Screenshot of GTEx database for significant SNP pair rs7107152 and rs1242229. Figure S2. Screenshot of NESDA NTR Conditional eQTL Catalog for rs7107152 and rs1242229. Figure S3. Screenshot of RegulomeDB for rs1242229. Figure S4. Radar chart showing the result of the rs7107152 and rs1242229 SNP pair–single trait (metabolic syndrome-component trait) association analysis. Figure S5. Screenshot from the GTEx database of the eQTL SNP-enriched region around SIDT2 and TAGLN (chr11:117,000,000-117,100,000). Figure S6 Model for how significant SNP pair rs7107152/rs1242229 may affect metabolic syndrome risk. Table S1. Characteristics of gene-based SNPs set. Table S2. Minor allele frequency of identified SNPs. (PDF 975 kb) [file 40246_2018_180_MOESM1_ESM.pdf]

Additional file1

Multiple genotype-phenotype association study reveals intronic variant pair on *SIDT2* associated with metabolic syndrome in a Korean population

Sanghoon Moon[1‡], Young Lee[1,2‡], Sungho Won[3], Juyoung Lee[1*]

[1]Division of Genome Research, Center for Genome Science, Korea National Institute of Health, Chungcheongbuk-do, Korea

[2]Veterans Medical Research Institute, Veterans Health Service Medical Center, Seoul, Korea

[3]Department of Public Health Science, Seoul National University, Seoul, Korea

*Correspondence to: Juyoung Lee, PhD, Email: jylee@cdc.go.kr

Supplementary Figure legends

Figure S1. (A, B) Screenshot of GTEx database for significant SNP pair rs7107152 and rs1242229. Gene expression P-values of rs1242229 (A) and rs7107152 (B) for *SIDT2* and *TAGLN* in whole blood are shown. (C–F) Gene expression analysis by genotype of rs7107152 for *SIDT2* (C) and *TAGLN* (D) and of rs1242229 for *SIDT2* (E) and *TAGLN* (F). The graphics and gene models included in this figure were made based on the UCSC genome browser.

Figure S2. (A, B) Screenshot of NESDA NTR Conditional eQTL Catalog for rs7107152 (A) and rs1242229 (B).

Figure S3. Screenshot of RegulomeDB for rs1242229.

Figure S4. Radar chart showing the result of the rs7107152 and rs1242229 SNP pair–single trait (metabolic syndrome-component trait) association analysis.

Figure S5. Screenshot from the GTEx database of the eQTL SNP–enriched region around *SIDT2* and *TAGLN* (chr11:117,000,000–117,100,000). Color dots below rs7107152 means statistical significant of eQTL for corresponding gene

Figure S6. Model for how significant SNP pair rs7107152/rs1242229 may affect metabolic syndrome risk

Figure S1.

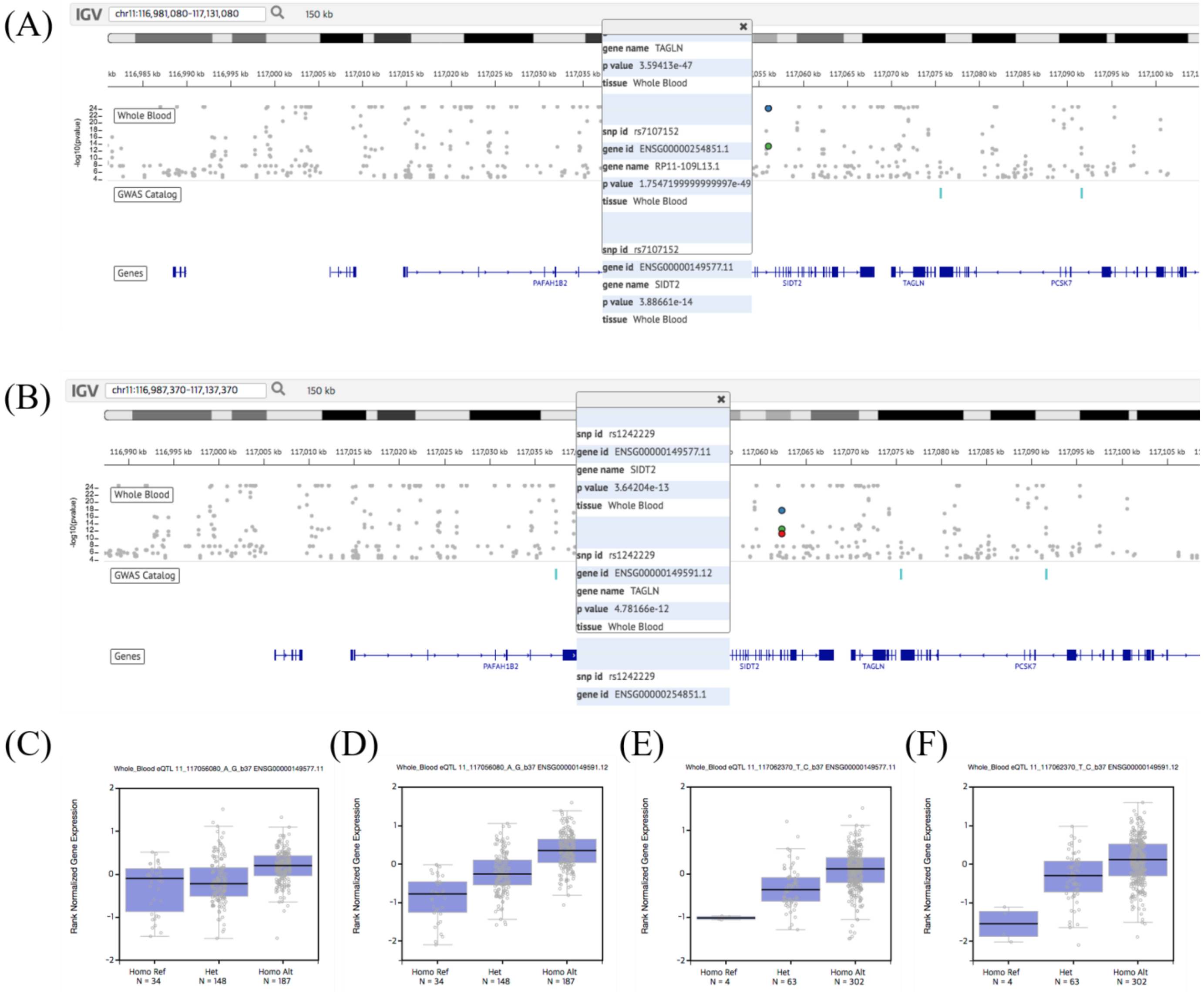
(A)
IGV
chr11:116,981,080-117,131,080
150 kb
Whole Blood
GWAS Catalog
Genes
-log10(pvalue)
gene name TAGLN
p value 3.59413e-47
tissue Whole Blood
snp id rs7107152
gene id ENSG00000254851.1
gene name RP11-109L13.1
p value 1.7547199999999997e-49
tissue Whole Blood
snp id rs7107152
gene id ENSG00000149577.11
gene name SIDT2
p value 3.88661e-14
tissue Whole Blood
PAFAH1B2
SIDT2
TAGLN
PCSK7
(B)
IGV
chr11:116,987,370-117,137,370
150 kb
Whole Blood
GWAS Catalog
Genes
-log10(pvalue)
snp id rs1242229
gene id ENSG00000149577.11
gene name SIDT2
p value 3.64204e-13
tissue Whole Blood
snp id rs1242229
gene id ENSG00000149591.12
gene name TAGLN
p value 4.78166e-12
tissue Whole Blood
snp id rs1242229
gene id ENSG00000254851.1
PAFAH1B2
SIDT2
TAGLN
PCSK7
(C)
Whole_Blood eQTL 11_117056080_A_G_b37 ENSG00000149577.11
Rank Normalized Gene Expression
Homo Ref N = 34
Het N = 148
Homo Alt N = 187
(D)
Whole_Blood eQTL 11_117056080_A_G_b37 ENSG00000149591.12
Rank Normalized Gene Expression
Homo Ref N = 34
Het N = 148
Homo Alt N = 187
(E)
Whole_Blood eQTL 11_117062370_T_C_b37 ENSG00000149577.11
Rank Normalized Gene Expression
Homo Ref N = 4
Het N = 63
Homo Alt N = 302
(F)
Whole_Blood eQTL 11_117062370_T_C_b37 ENSG00000149591.12
Rank Normalized Gene Expression
Homo Ref N = 4
Het N = 63
Homo Alt N = 302

Figure S2.

(A)

NESDA NTR Conditional eQTL Catalog

Search Genes (Cis) Genes (Trans) Download Info

cis eQTL Details

Text-Only Results

Conditional on #SNPs	SNP ID	SNP ID RSID	Probeset ID	Gene	Beta	P-value	FDR
0	11:117056080_A	rs7107152	11753063_a_at	SIDT2	-0.682	1.090000e-260	<1.34e-05
0	11:117056080_A	rs7107152	11717990_a_at	SIDT2	-0.451	7.160000e-103	<1.34e-05
0	11:117056080_A	rs7107152	11717991_a_at	SIDT2	-0.353	1.160000e-55	<1.34e-05
0	11:117056080_A	rs7107152	11754096_x_at	TAGLN	-0.18	2.060000e-15	<1.34e-05
0	11:117056080_A	rs7107152	11749922_x_at	TAGLN	-0.156	4.090000e-12	<1.34e-05
0	11:117056080_A	rs7107152	11754095_a_at	TAGLN	-0.15	1.980000e-11	<1.34e-05
0	11:117056080_A	rs7107152	11746699_x_at	TAGLN	-0.135	4.580000e-9	6.57e-05
0	11:117056080_A	rs7107152	11726604_a_at	PAFAH1B2	-0.123	8.170000e-8	7.44e-04
0	11:117056080_A	rs7107152	11753627_x_at	TAGLN	-0.114	1.220000e-6	8.04e-03
0	11:117056080_A	rs7107152	11731978_s_at	PCSK7	0.096	8.480000e-6	4.31e-02
1	11:117056080_A	rs7107152	11758962_at	PAFAH1B2	0.114	7.300000e-8	6.31e-04
1	11:117056080_A	rs7107152	11758964_x_at	PAFAH1B2	0.098	2.980000e-6	1.74e-02

Total found: **12** Records

(B)

NESDA NTR Conditional eQTL Catalog

Search Genes (Cis) Genes (Trans) Download Info

cis eQTL Details

Text-Only Results

Conditional on #SNPs	SNP ID	SNP ID RSID	Probeset ID	Gene	Beta	P-value	FDR
0	11:117062370_T	rs1242229	11753063_a_at	SIDT2	-0.752	2.270000e-155	<1.34e-05
0	11:117062370_T	rs1242229	11717990_a_at	SIDT2	-0.476	2.950000e-58	<1.34e-05
0	11:117062370_T	rs1242229	11717991_a_at	SIDT2	-0.388	9.050000e-35	<1.34e-05
0	11:117062370_T	rs1242229	11754096_x_at	TAGLN	-0.219	5.200000e-12	<1.34e-05
0	11:117062370_T	rs1242229	11754095_a_at	TAGLN	-0.205	4.890000e-11	<1.34e-05
0	11:117062370_T	rs1242229	11749922_x_at	TAGLN	-0.154	9.790000e-7	6.43e-03

Total found: **6** Records

Figure S3.

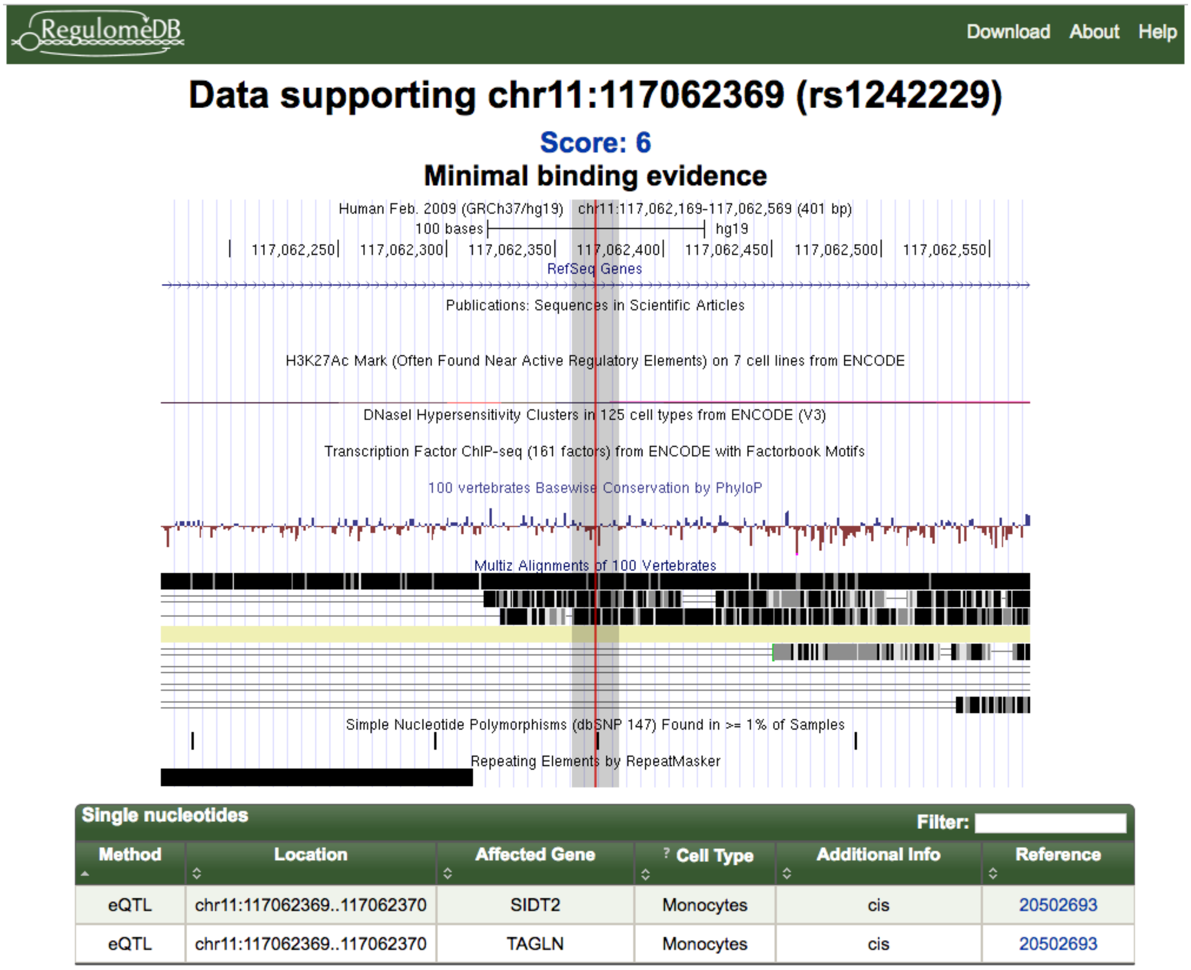
RegulomeDB
Download
About
Help
Data supporting chr11:117062369 (rs1242229)
Score: 6
Minimal binding evidence
Human Feb. 2009 (GRCh37/hg19) chr11:117,062,169-117,062,569 (401 bp)
100 bases
hg19
117,062,250
117,062,300
117,062,350
117,062,400
117,062,450
117,062,500
117,062,550
RefSeq Genes
Publications: Sequences in Scientific Articles
H3K27Ac Mark (Often Found Near Active Regulatory Elements) on 7 cell lines from ENCODE
DNaseI Hypersensitivity Clusters in 125 cell types from ENCODE (V3)
Transcription Factor ChIP-seq (161 factors) from ENCODE with Factorbook Motifs
100 vertebrates Basewise Conservation by PhyloP
Multiz Alignments of 100 Vertebrates
Simple Nucleotide Polymorphisms (dbSNP 147) Found in >= 1% of Samples
Repeating Elements by RepeatMasker
Single nucleotides
Filter:
Method
Location
Affected Gene
Cell Type
Additional Info
Reference
eQTL
chr11:117062369..117062370
SIDT2
Monocytes
cis
20502693
eQTL
chr11:117062369..117062370
TAGLN
Monocytes
cis
20502693

Figure S4.

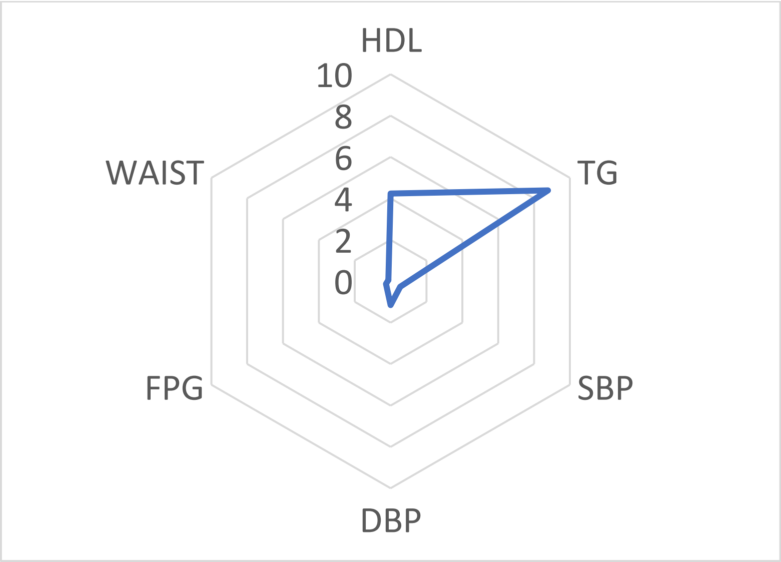

Figure S5.

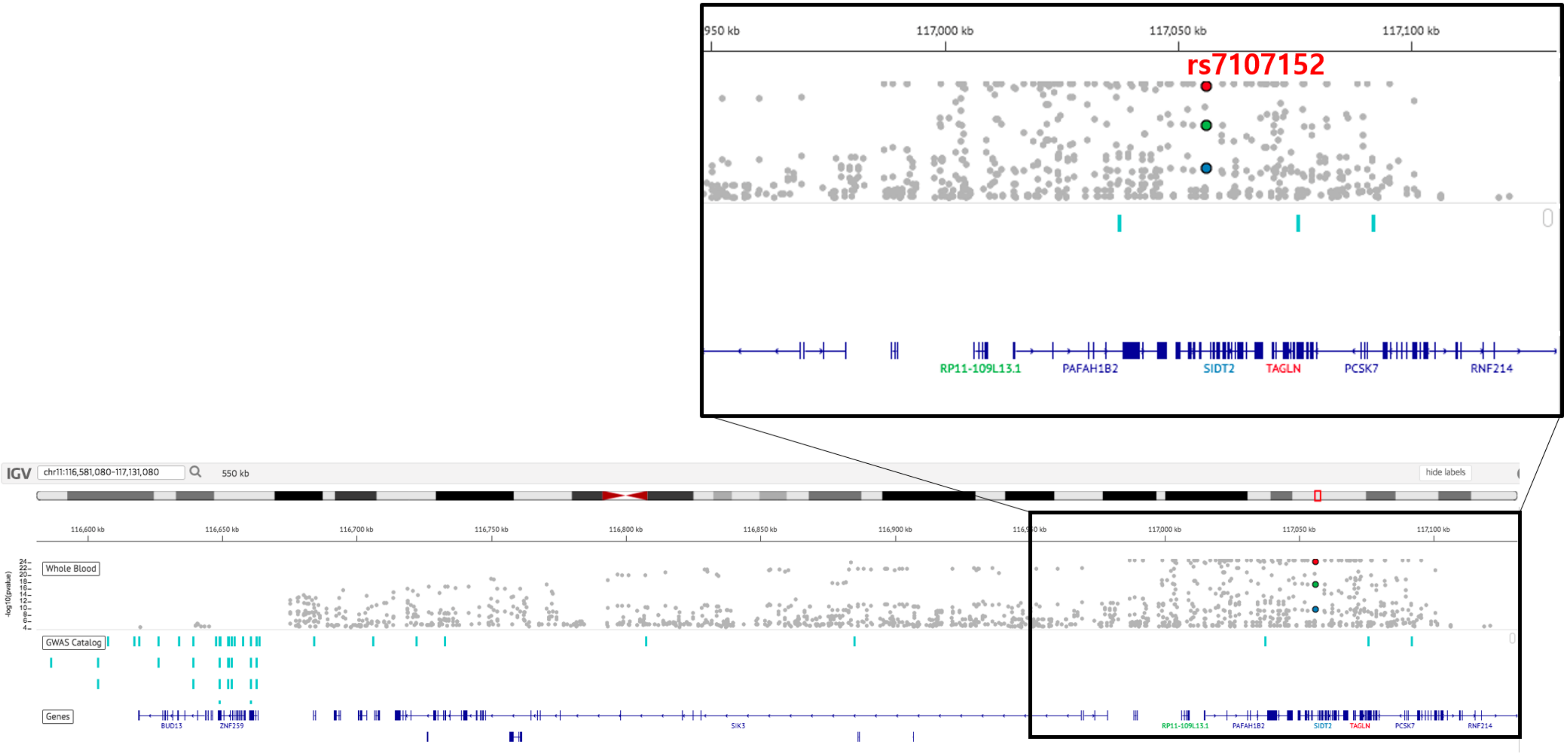
950 kb
117,000 kb
117,050 kb
117,100 kb
rs7107152
RP11-109L13.1
PAFAH1B2
SIDT2
TAGLN
PCSK7
RNF214
IGV
chr11:116,581,080-117,131,080
550 kb
hide labels
116,600 kb
116,650 kb
116,700 kb
116,750 kb
116,800 kb
116,850 kb
116,900 kb
117,000 kb
117,050 kb
117,100 kb
Whole Blood
-log10(pvalue)
GWAS Catalog
Genes
BUD13
ZNF259
SIK3

Figure S6.

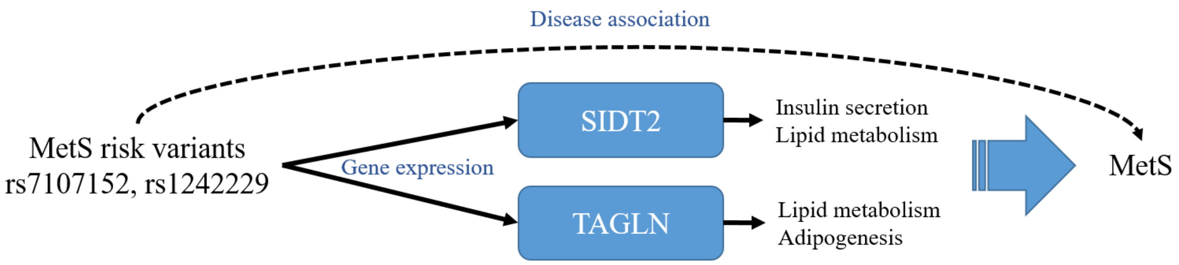
Disease association
MetS risk variants
rs7107152, rs1242229
Gene expression
SIDT2
TAGLN
Insulin secretion
Lipid metabolism
Lipid metabolism
Adipogenesis
MetS

Table S1. Characteristics of gene-based SNPs set.

Total number of gene-based set	14475
Number of set which is consist of one SNP	4414
1st quantile of number of set	1
Median of number of set	3
Mean of number of set	6.895
3rd quantile of number of set	5
Maximum size of set	3300

Table S2. Minor allele frequency of identified SNPs.

rs_number	Affymetrix SNP ID	CHR	A1	A2	MAF	NCHROBS
rs2293571	SNP_A-4261809	2	T	C	0.125	14418
rs780094	SNP_A-1909462	2	C	T	0.460	14414
rs780092	SNP_A-2094945	2	G	A	0.323	14414
rs8179252	SNP_A-2045362	2	C	A	0.132	14356
rs263	SNP_A-2309257	8	A	G	0.210	14422
rs271	SNP_A-2303801	8	T	C	0.210	14422
rs12545984	SNP_A-2077138	8	A	G	0.084	14422
rs10503669	SNP_A-4212851	8	T	G	0.122	14256
rs17410962	SNP_A-2305697	8	A	G	0.126	14412
rs17489282	SNP_A-4246847	8	A	G	0.208	14260
rs4922117	SNP_A-1783370	8	G	A	0.209	14352
rs765547	SNP_A-1809373	8	T	C	0.210	14420
rs11986942	SNP_A-2255525	8	C	G	0.210	14414
rs1837842	SNP_A-2072422	8	C	T	0.209	14402
rs1919484	SNP_A-1961393	8	T	C	0.209	14416
rs7461115	SNP_A-2141946	8	C	G	0.206	14422
rs7013777	SNP_A-2068733	8	G	A	0.209	14416
rs4442164	SNP_A-1860217	8	G	A	0.076	14290
rs4244457	SNP_A-2013262	8	T	C	0.326	14388
rs4449813	SNP_A-1834498	8	A	G	0.068	14420
rs12686004	SNP_A-2022938	9	T	C	0.212	14418
rs3905000	SNP_A-4267633	9	A	G	0.057	14272
rs481843	SNP_A-2201273	11	T	C	0.111	14420
rs486394	SNP_A-2193815	11	C	A	0.122	14422
rs180344	SNP_A-4243521	11	G	A	0.309	14350
rs11216126	SNP_A-1864563	11	C	A	0.204	14412
rs6589566	SNP_A-4214168	11	C	T	0.219	14394
rs603446	SNP_A-2138134	11	T	C	0.225	14420
rs11600380	SNP_A-4270799	11	C	T	0.044	14406
rs6589567	SNP_A-4248377	11	T	G	0.274	14420
rs12279433	SNP_A-1936154	11	T	G	0.098	14418
rs11827828	SNP_A-2273692	11	A	G	0.068	14422
rs2044426	SNP_A-2302239	11	T	C	0.098	14422
rs10892044	SNP_A-2130520	11	C	T	0.068	14420
rs11216186	SNP_A-1841925	11	G	A	0.126	14418
**rs7107152**	**SNP_A-4270800**	**11**	**C**	**T**	**0.359**	**14064**
**rs1242229**	**SNP_A-1876133**	**11**	**A**	**G**	**0.448**	**14322**
rs10892876	SNP_A-2293097	11	T	C	0.164	14422
rs12290043	SNP_A-1891245	11	A	C	0.164	14420
rs12420127	SNP_A-4291073	11	G	A	0.126	14028
rs10790175	SNP_A-2029222	11	G	A	0.360	14324
rs10892082	SNP_A-2038801	11	A	C	0.456	14418
rs1784042	SNP_A-2169941	11	T	C	0.173	14420
rs2074356	SNP_A-1796043	12	T	C	0.149	14420
rs11066194	SNP_A-2088716	12	A	G	0.391	14422
rs886126	SNP_A-2098665	12	C	T	0.372	14312
rs2078851	SNP_A-2298368	12	C	T	0.466	14422
rs7952972	SNP_A-4288051	12	T	A	0.403	14240
rs7300082	SNP_A-2239662	12	G	A	0.169	14404
rs4766553	SNP_A-2037268	12	C	A	0.494	14422
rs1265566	SNP_A-2097940	12	G	A	0.325	14422
rs9783423	SNP_A-1811058	12	C	G	0.291	14402
rs7398833	SNP_A-2298474	12	T	C	0.059	14422
rs16941414	SNP_A-2310909	12	A	G	0.136	14420
rs6489979	SNP_A-1832759	12	A	G	0.267	14324
rs16941284	SNP_A-2256250	12	A	T	0.094	14364
rs16941319	SNP_A-4277711	12	C	G	0.057	14418
rs11065851	SNP_A-4291625	12	A	G	0.048	14422

rs756825	SNP_A-2174172	12	G	A	0.482	14418
rs7300860	SNP_A-1860098	12	T	C	0.146	14420
rs11631342	SNP_A-4271137	15	G	A	0.065	14420
rs6494005	SNP_A-2034106	15	G	A	0.269	14312

CHR: chromosome; A1: Allele 1 code (minor allele); A2: Allele 2 code (major allele); MAF: Minor allele frequency; NCHROBS: Non-missing allele count. SNPs on *SIDT2* are shown in boldface.
